# Pharmacokinetic Modeling of the Effect of Tariquidar on Ondansetron Disposition into the Central Nervous System

**DOI:** 10.1007/s11095-024-03739-6

**Published:** 2024-07-09

**Authors:** Manting Chiang, Hyunmoon Back, Jong Bong Lee, Sarah Oh, Tiffany Guo, Simone Girgis, Celine Park, Simon Haroutounian, Leonid Kagan

**Affiliations:** 1 Department of Pharmaceutics, Ernest Mario School of Pharmacy, Rutgers, The State University of New Jersey, 160 Frelinghuysen Road, Piscataway, NJ 08854 USA; 2 Division of Clinical and Translational Research and Washington University Pain Center, Department of Anesthesiology, Washington University School of Medicine, St Louis, MO USA; 3 Center of Excellence for Pharmaceutical Translational Research and Education, Ernest Mario School of Pharmacy, Rutgers, The State University of New Jersey, Piscataway, NJ USA

**Keywords:** biodisposition, brain, neuropathic pain, P-glycoprotein

## Abstract

**Purpose:**

Serotonin (5-HT_3_) receptor antagonists are promising agents for treatment of neuropathic pain. However, insufficient drug exposure at the central nervous system (CNS) might result in lack of efficacy. The goal of this study was to evaluate the impact of administration of a Pgp inhibitor (tariquidar) on ondansetron exposure in the brain, spinal cord, and cerebrospinal fluid in a wild-type rat model.

**Methods:**

Ondansetron (10 mg/kg) and tariquidar (7.5 mg/kg) were administered intravenously, plasma and tissue samples were collected and analyzed by HPLC. A mathematical model with brain, spinal cord, cerebrospinal fluid and two systemic disposition compartments was developed to describe the data.

**Results:**

The results demonstrate that tariquidar at 7.5 mg/kg resulted in a complete inhibition of Pgp efflux of ondansetron in the brain and spinal cord. The compartmental model successfully captured pharmacokinetics of ondansetron in wild type and Pgp knockout (KO) animals receiving the drug alone or in wild type animals receiving the ondansetron and tariquidar combination.

**Conclusions:**

The study provided important quantitative information on enhancement of CNS exposure to ondansetron using co-administration of Pgp Inhibitor in a rat model, which will be further utilized in conducting a clinical study. Tariquidar co-administration resulted in ondansetron CNS exposure comparable to observed in Pgp KO rats. Results also highlighted the effect of tariquidar on plasma disposition of ondansetron, which may not be dependent on Pgp inhibition, and should be evaluated in future studies.

## Introduction

Neuropathic pain is characterized by a lesion- or disease-related changes in nociceptive signal transmission or processing in the nervous system [1]. Mechanistically, neuropathic pain involves altered electrical properties of afferent fibers carrying nociceptive information, but also involves changes in the spinal and supraspinal somatosensory pathways [1]. The spinal cord acts as the main site of signal integration, where descending pathways from cortical regions can modulate the excitatory and inhibitory activity of interneurons and glial cells on incoming signals from peripheral afferent fibers [1]. Extensive research has been done to understand how these descending control mechanisms may behave under normal conditions and in the context of nerve injury [1, 2].

Although the serotonergic descending pathways are typically referred to as pain inhibitory, they can have both inhibitory and excitatory properties under different conditions [2]. In the context of peripheral nerve injury, an increase in excitatory activity has been shown through the activity of the serotonin receptor subtype 3 (5-HT_3_) receptors in the spinal cord [3–5]. Additional evidence also links accelerated turnover of serotonin (5-HT) in the spinal cord [6, 7]. Interest in the administration of 5-HT_3_ receptor antagonists to treat neuropathic pain has grown, with both preclinical and clinical reports investigating the pain relief potential of 5-HT_3_ receptor antagonists such as ondansetron and tropisetron, commonly used for antiemetic treatment. Intrathecal administration of ondansetron in a neuropathic animal model demonstrated a dose-dependent antinociceptive effect [8]. However, previously published clinical reports have been inconclusive, with some studies reporting effective pain relief following a single IV dose of 8 mg ondansetron, while others report no pain improvement compared to placebo [9, 10]. The inconclusive clinical reports may be related to insufficient drug exposure at the target (i.e., spinal 5-HT_3_ receptors) rather than ineffective pharmacological approach.

Achieving sufficient drug concentrations in the central nervous system (CNS) is crucial for therapeutic efficacy of centrally-acting drugs. Active drug efflux by P-glycoprotein (Pgp) transporters at the blood–brain barrier (BBB) has shown a significant impact in limiting CNS exposure to a wide range of compounds that are substrates for Pgp [11, 12]. Specific inhibitors to Pgp have been evaluated following co-administration with Pgp substrates to achieve target concentrations at the site of action [13, 14]. Tariquidar (XR9567) is a third-generation Pgp inhibitor that has demonstrated potent inhibitory activity (nM-range IC_50_) and affinity to Pgp (K_D_ = 5.1 nM) [15–17].

Ondansetron is subject to Pgp efflux at BBB, which limits its penetration into the CNS. In our previous study, Pgp knock-out rats demonstrated significantly higher exposure of ondansetron in the brain, spinal cord, and cerebrospinal fluid, compared to wild-type rats when administered a single IV bolus dose of ondansetron (10 mg/kg) [18]. However, plasma concentrations did not differ between wild-type and Pgp knock-out rats. These findings are corroborated by another report [11].

The goal of this study was to evaluate the impact of tariquidar administration on ondansetron exposure at different regions of the CNS (brain, spinal cord, and cerebrospinal fluid) in a wild-type rat model. This study built upon our previously obtained results using a genetic knockout rat model [18] and served as a preclinical proof of principle for a currently ongoing clinical study. Another goal was to investigate differences in tariquidar effects on ondansetron biodisposition between male and female rats. A mechanism-based model was developed to capture systemic pharmacokinetics and CNS disposition of ondansetron in all groups.

## Materials and Methods

### Materials

Ondansetron hydrochloride, *N*-benzylbenzamide, ethylenediaminetetraacetic acid tripotassium (K_3_EDTA) were obtained from Sigma-Aldrich (St. Louis, MO, USA). Tariquidar was obtained from Azatrius Pharmaceuticals (Mumbai, India). Pooled rat plasma and cerebrospinal fluid (CSF) was purchased from BioIVT (Westbury, NY, USA). All solvents utilized for the experiments were purchased as HPLC grade or higher from Fisher Scientific (Fair Lawn, NJ, USA).

### Animals

The study was conducted under the protocol approved by the Institutional Animal Care and Use Committee at Rutgers University. Wild-type Sprague Dawley rats (males of 10–12 weeks, weighing 300–350 g and females of 12–14 weeks, weighing 250–280 g) were purchased from Horizon Discovery (previously Sage Labs Inc., Boyertown, PA). Animals were housed in a vivarium under controlled temperatures and 12/12 h dark/light cycle with free access to food and water. Right jugular vein was cannulated to allow for accurate intravenous dosing using polyethylene tubing (PE-50, Braintree Scientific, Braintree, MA). The surgery was performed under light isoflurane anesthesia, and after that animals were allowed to recover for 24 h. Intradermal bupivacaine and subcutaneous meloxicam were provided as analgesia.

### Experimental Design

Initially, effect of tariquidar on systemic disposition of ondansetron was evaluated in a single dose study with sequential blood sampling in male (OT-M) and female (OT-F) rats. Ondansetron (10 mg/kg, 10 mg/mL in water) and tariquidar (7.5 mg/kg, 5 mg/mL in 2% aqueous dextran solution) were administered through the jugular catheter followed by a saline flush (0.2 mL). The saline flush ensured that there was no drug contamination in the catheter, and it was suitable for blood sampling (as was confirmed in our previous studies). Tariquidar formulation preparation and dose selection followed a previous preclinical report [19], and it was administered 30 min prior to ondansetron. Following ondansetron administration, serial blood samples (250 µL) were collected at 5, 10, 15, 30, 45 min, 1, 1.5, 2, 2.5, 3, and 4 h into EDTA tubes. Heparinized saline (20 IU/mL) was used for volume replacement after each sample. Plasma was separated by centrifugation (7 min, 13,000 RPM), transferred to a fresh tube, and stored at −20°C pending analysis.

At the next stage, effect of Pgp inhibition using tariquidar on CNS disposition of ondansetron was evaluated. Animals previously used in the plasma pharmacokinetics study were included following a two-week washout period; and additional animals were added to achieve *n* = 4–6 per time point. Tariquidar solution (7.5 mg/kg) followed after 30 min by ondansetron solution (10 mg/kg) were injected intravenously through the jugular vein cannula or through the tail vein. Groups of rats were sacrificed at 0.16, 0.25, 0.5, 1, 1.5, 2, and 2.5 h under isoflurane anesthesia, and terminal samples were collected including plasma, brain, spinal cord, and cerebrospinal fluid from cisterna magna (CSF) and stored at −20°C.

### Sample Analysis

Agilent 1260 Infinity HPLC equipped with a photodiode array detector was used (Agilent, Santa Clara, CA, USA) in this work. The approach for analysis of ondansetron and tariquidar in plasma and tissue was based on our published methods [18, 20]. The limit of detection for ondansetron was 10 ng/mL for plasma and 50 ng/mL for CNS tissues. The limit of detection for tariquidar was 50 ng/mL for plasma and CSF and 100 ng/mL for brain and spinal cord samples. Inter-day and intra-day accuracy and precision of the assay were within 15%.

### Data Analysis

Ondansetron and tariquidar concentrations in were reported as mean ± SD for each time point. Data analysis was performed separately for male (OT-M) and female (OT-F) rat groups. Furthermore, ondansetron concentration in plasma and tissues (and corresponding pharmacokinetic parameters) were compared to the results of our previous study, in which ondansetron was administered alone as a 10 mg/kg single intravenous bolus to male and female wild type (WT-M, WT-F) and Pgp knockout (KO-M, KO-F) animals [18].

A standard noncompartmental analysis was performed using average plasma concentration–time data (samples obtained from sequential and terminal experiments were combined) in Phoenix WinNonlin version 8.1 (Certara, Princeton, NJ). For plasma, the area under the plasma concentration–time curve from time zero to infinity (AUC_plasma_), systemic clearance (CL), volume of distribution at steady state (V_d,ss_), half-life (t_1/2,plasma_), and mean residence time (MRT) were calculated. For brain, spinal cord, and CSF the area under the tissue concentration–time curve (AUC_tissue_) and half-life in tissue (t_1/2,tissue_) were reported.

Ondansetron partition coefficient (Kp) for each of the tested CNS compartments was calculated as a ratio between the AUC_tissue_ and the AUC_plasma_. However, since the AUC values were based on terminal samples, no statistical comparison among the groups could be performed. The effect of tariquidar-mediated Pgp inhibition on ondansetron exposure was further evaluated by comparing partition coefficients calculated in this study with partition coefficients in WT and Pgp knockout (KO) rats from our previous study [18].

### Pharmacokinetic Modeling

**Fig. 1:**
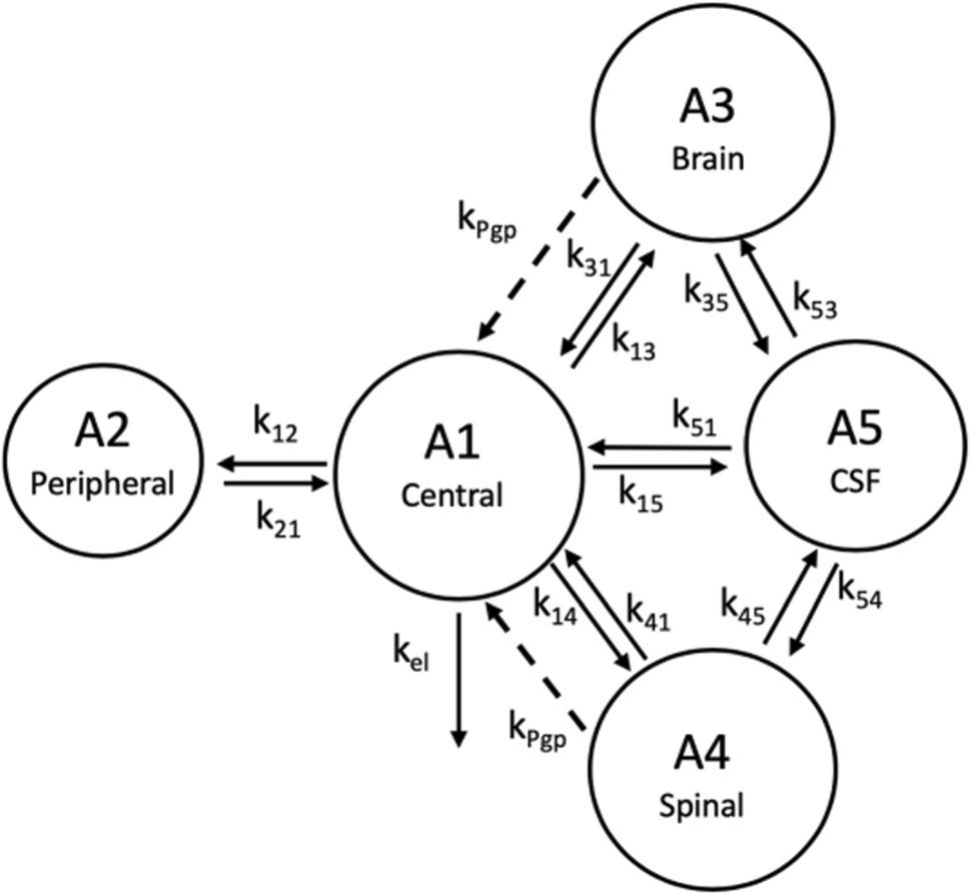
Schematic of the semi-physiological pharmacokinetic model used to capture systemic disposition and CNS distribution of ondansetron in male and female wild type and Pgp knockout rats after administration of ondansetron alone and in wild type rats following co-dosing with tariquidar. Model equations and parameters are described in Methods and Table III. k_Pgp_ was included only for wild type animals.

The pharmacokinetic model developed previously to describe ondansetron systemic disposition and distribution into various parts of the CNS in wild-type and Pgp knock-out rats was adopted for this analysis [18]. The model schematic is shown in Fig. 1. Briefly, the systemic disposition of ondansetron is described by a central (A_1_) and peripheral (A_2_) distribution compartments and a linear elimination process (k_el_). Interconnectivity of CNS compartments followed animal physiology (A3 - brain, A4 – spinal cord, A5 - CSF), and previously published CNS model were consulted [21–23]. The volume of the brain (V_brain_) was fixed to a previously determined value of 1.8 mL which was also in agreement with the brain weight reported before [18, 24, 25]. The volume of the CSF compartment (V_CSF_) was fixed to 0.25 mL [25]. The volume of the spinal cord (V_spinal_) was measured and reported in our previous study and was fixed to 0.6 mL. First order rate constant were used to describe a bidirectional passive permeability between the central disposition compartment (with the volume V_1_) and various CNS compartments (k_13_, k_31_, k_14_, k_41_, k_15_, k_51_) and distributional terms between CNS compartments (k_35_, k_53_, k_45_, k_54_). Pgp efflux at the BBB was described using a first-order term (k_pgp_) and was included for wild type animals only; the term was set to zero in KO rats. The following differential Eqs. (1–5) were used:

1 $$\frac{{dA}_{1}}{dt}={k}_{21}\cdot {A}_{2}+{k}_{31}\cdot {A}_{3}+{ k}_{41}\cdot {A}_{4}+{k}_{51}\cdot {A}_{5}-\left({k}_{el}+{k}_{12}+{k}_{13}+{k}_{14}+{k}_{15}\right)\cdot{A}_{1}+{k}_{Pgp}\cdot {A}_{3}+{k}_{Pgp}\cdot {A}_{4}$$

2 $$\frac{{dA}_{2}}{dt}={k}_{12}\cdot {A}_{1}-{k}_{21}\cdot {A}_{2}$$

3 $$\frac{{dA}_{3}}{dt}={k}_{13}\cdot {A}_{1}+{k}_{53}\cdot {A}_{5}-\left({k}_{31}+{k}_{35}+{k}_{Pgp}\right)\cdot {A}_{3}$$

4 $$\frac{{dA}_{4}}{dt}={k}_{14}\cdot {A}_{1}+{k}_{54}\cdot {A}_{5}-\left({k}_{41}+{k}_{45}+{k}_{Pgp}\right)\cdot {A}_{4}$$

5 $$\frac{{dA}_{5}}{dt}={k}_{15}\cdot {A}_{1}+{k}_{35}\cdot {A}_{3}+{k}_{45}\cdot {A}_{4}-\left({k}_{51}+{k}_{53}+{k}_{54}\right)\cdot {A}_{5}$$

The whole dataset included mean ondansetron profiles of OT-M and OT-F in plasma, brain, spinal cord, and CSF obtained in this study and previous data for male and female WT and KO animals. Due to changes in the plasma pharmacokinetics of ondansetron following co-administration of tariquidar separate terms for central volume of distribution(V_1,TRQ_) and elimination rate constant (k_el,TRQ_) were required to capture the data. Several functions to describe tariquidar-induced Pgp inhibition were considered. However, due to a long half-life of tariquidar observed in the study there was not enough information in the dataset to capture tariquidar concentration-dependent inhibition. The final model included a separate term for describing Pgp function in OT groups (k_Pgp,TRQ_). Other parameters were shared across WT, KO, and OT models. All model parameters were estimated. A separate set of parameters was estimated for female cohorts (WT-F, KO-F, OT-F) using the same final model structure.

Following establishing the final model, simulations were performed to assess the effect of the extent of Pgp inhibition on ondansetron exposure in the CNS. The value of K_Pgp_ was varied and the rest of the parameters were kept fixed to the previously estimated values.

R (version 3.31) and Rstudio (version 1.2.5001, Boston, MA, USA) with Ubiquity package were used for model development [26] as described before [18].

## Results

**Fig. 2:**
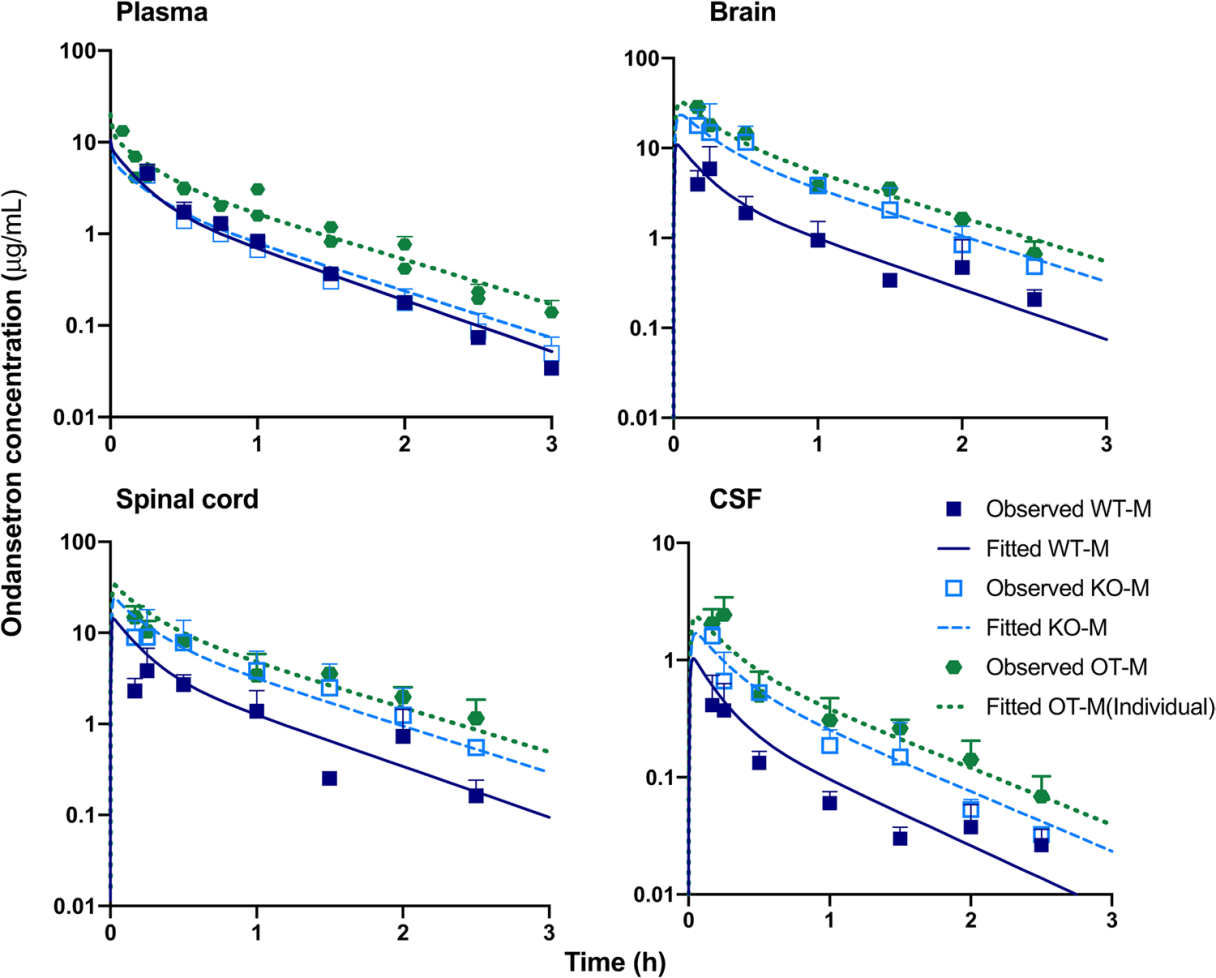
The observed (symbols) and fitted (lines) pharmacokinetic profiles of ondansetron in male rat (OT-M) following co-administration of ondansetron (10 mg/kg) and tariquidar (7.5 mg/kg) IV bolus and in male WT and KO rats following IV bolus 10 mg/kg dose of ondansetron alone. Observed data are shown as mean ± SD. Observed data for WT and KO animals were taken from the previous study [18].

**Fig. 3:**
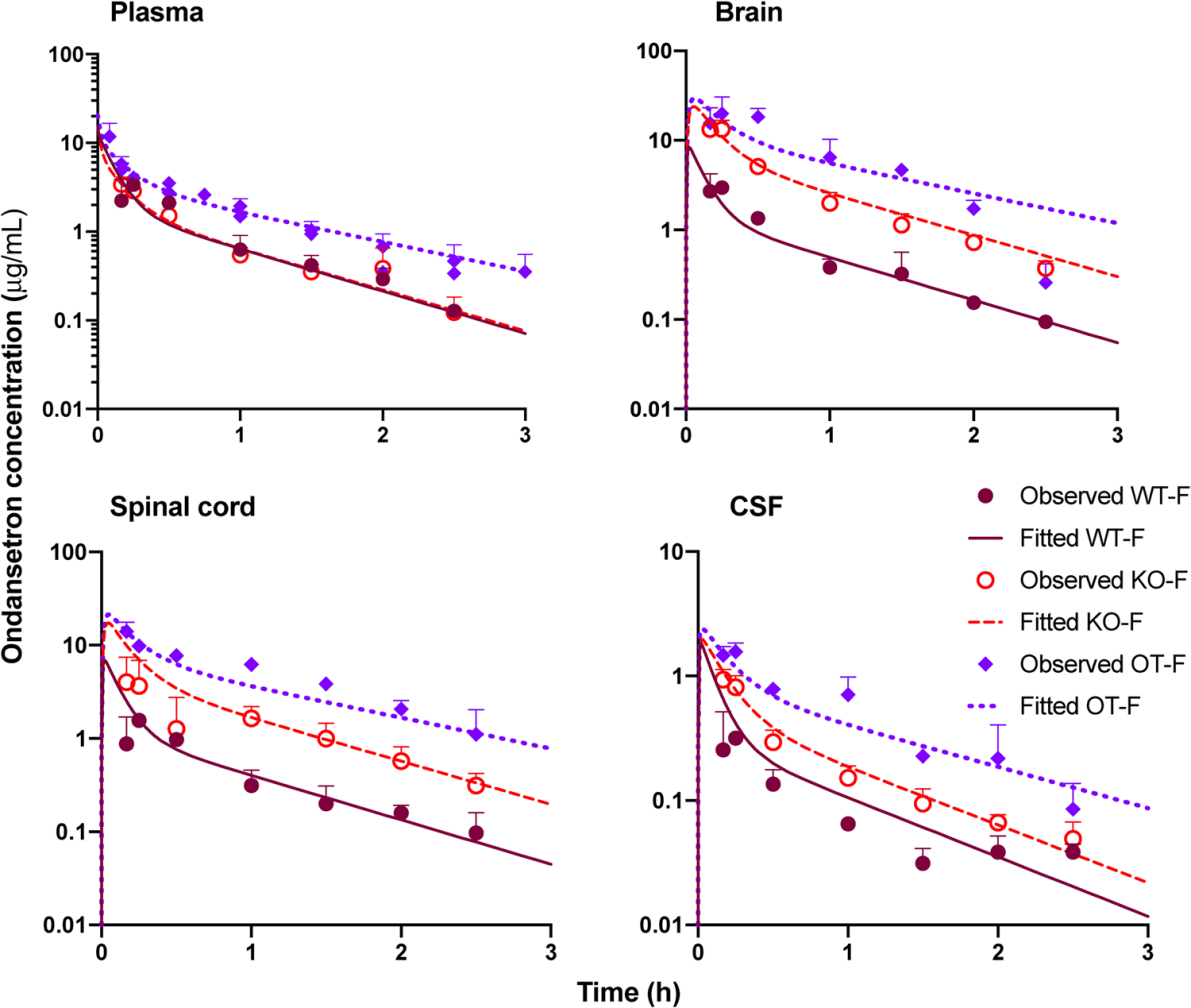
The observed (symbols) and fitted (lines) pharmacokinetic profiles of ondansetron in female rat (OT-F) following co-administration of ondansetron (10 mg/kg) and tariquidar (7.5 mg/kg) IV bolus and in female WT and KO rats following IV bolus 10 mg/kg dose of ondansetron alone. Observed data are shown as mean ± SD. Observed data for WT and KO animals were taken from the previous study [18].

The observed concentration–time profiles of ondansetron in plasma of OT-M and OT-F were overlaid for comparison with previous results from WT-M, KO-M and WT-F, KO-F animals. For both OT-M and OT-F, ondansetron plasma concentration was increased when compared to WT and KO animals (Figs. 2 and 3). The results of the noncompartmental analysis of plasma data are presented in Table I; and no statistical comparison could be conducted. The full PK study was conducted until 2.5 h due to the limitations of the bioanalytical limit of detection for ondansetron in the CNS. Comparison of noncompartmental parameters between sexes showed similarities between the two groups with plasma AUC of 6.13 and 5.65 μg⋅h⋅mL^−1^ respectively. These values were nearly double of what was found in WT and KO animals [18].

**Table I:** Noncompartmental Plasma Pharmacokinetic Parameters of Ondansetron Following Co-administration IV of Ondansetron (10 mg/kg) and Tariquidar (7.5 mg/kg)

Parameter	OT-M	OT-F
t_1/2,plasma_ (h)	0.638	0.998
AUC_plasma,0-∞_ (h ⋅μg⋅mL^−1^)	6.13	5.65
MRT (h)	0.577	0.919
V_d,ss_ (mL)	302	396
CL (mL⋅h^−1^)	525	447

The CNS tissue disposition study revealed that in both sexes co-administration with tariquidar resulted in higher concentration of ondansetron in all tested regions of the CNS as compared to both WT rat (administered ondansetron alone) and KO strain (Figs. 2 and 3). In the presence of tariquidar, ondansetron partition coefficients (K_P_OT_) ranged from 2.44 to 3.58 in the brain and spinal cord (Table II), which was considerably higher than without tariquidar in WT rats (K_P_WT_ range 0.39—0.71) and more similar to Pgp KO rats (K_P_KO_ range 1.34—3.04), as was determined in our previous study [18]. In the CSF, the partition coefficients for CSF were lower than in the brain or spinal cord (Table II); however, the values in the presence tariquidar were very similar to Pgp KO rats.

**Table II:** Noncompartmental Pharmacokinetic Parameters for Ondansetron in Brain, Spinal Cord and Cerebrospinal Fluid Following Co-administration IV of Ondansetron (10 mg/kg) and Tariquidar (7.5 mg/kg)

Parameter	Brain		Spinal Cord		CSF	
	OT-M	OT-F	OT-M	OT-F	OT-M	OT-F
t_1/2,tissue_ (h)	0.580	0.352	0.907	0.596	0.517	0.580
AUC_tissue,0-∞_ (h⋅μg⋅mL^−1^)	22.0	19.16	15.0	15.6	1.37	1.49
K_P_OT_	3.58	3.39	2.44	2.76	0.223	0.263
K_P_WT_*	0.46	0.71	0.39	0.47	0.06	0.11
K_P_KO_*	2.52	3.04	1.78	1.34	0.19	0.22

*OT* ondansetron and tariquidar co-administration group, *WT* ondansetron administered alone in wild type rats, *KO* ondansetron administered alone in Pgp knockout rats, *M* male rats, *F* female rats

^*^ data for WT and KO rats were extracted from the previous publication [18] for comparison

**Fig. 4:**
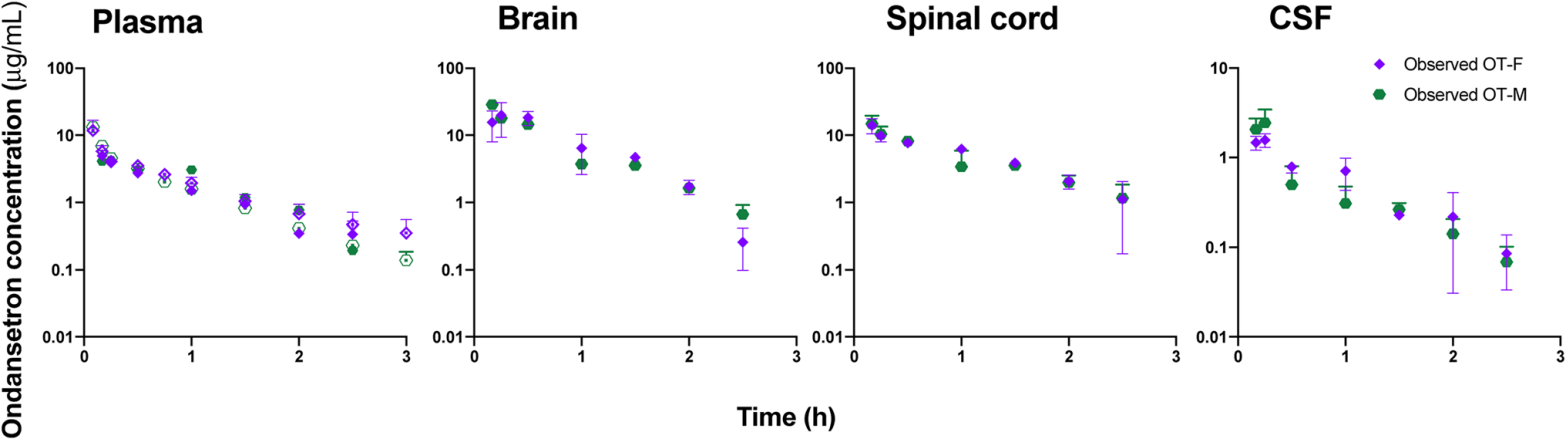
Observed pharmacokinetic profiles (mean ± SD) for OT-M and OT-F animals following IV bolus 10 mg/kg dose of ondansetron co-administered with 7.5 mg/kg tariquidar.

A comparison of pharmacokinetic profiles of ondansetron between the sexes is shown in Fig. 4. The observed OT-M and OT-F profiles are nearly super-imposed for plasma, brain, spinal cord, and CSF compartments.

**Fig. 5:**
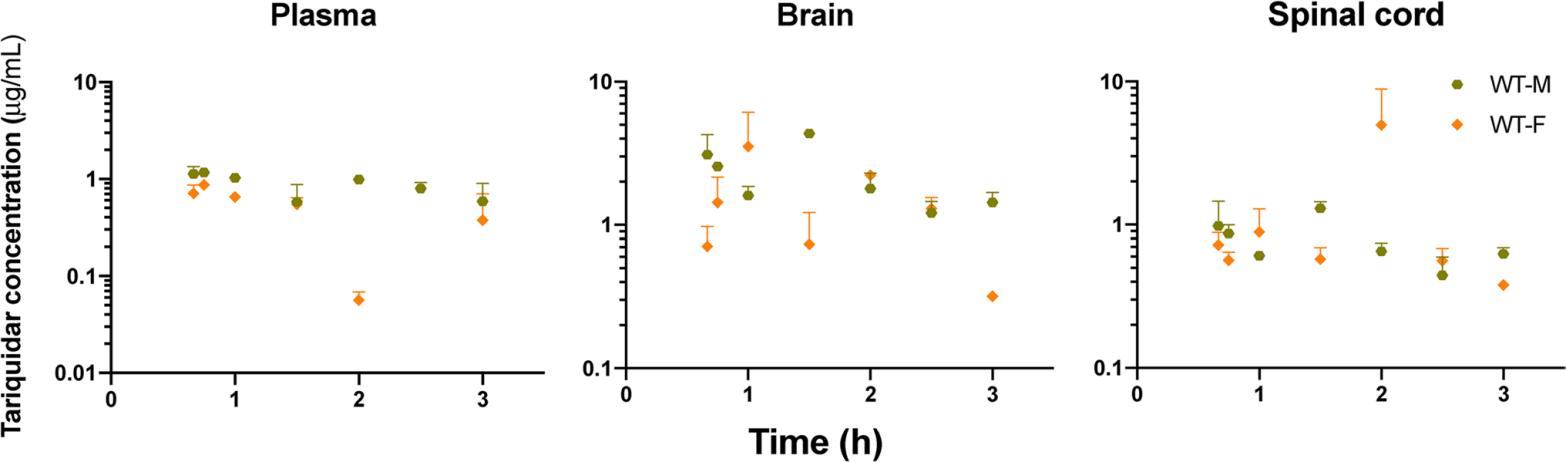
Observed tariquidar concentrations (mean ± SD) in plasma, brain and spinal cord following IV administration of 7.5 mg/kg to rats.

Tariquidar concentrations were also measured in all collected samples (Fig. 5). Relatively high variability between the animals was observed, and no sex-dependent trends in pharmacokinetics could be identified. Tariquidar could not be detected in any of the CSF samples with an established method and the limit of quantification. The half-life of tariquidar in plasma was 3.1 h in male and 2.2 h in female rats; similar long half-lives were observed in the brain and spinal cord. Tariquidar concentration in the brain was 2 to 3-fold higher than in plasma and exposure in the spinal cord was comparable to plasma. Because tariquidar concentration did not vary significantly over the time course of ondansetron pharmacokinetic experiment, presence of tariquidar was treated as a categorical covariate in the ondansetron pharmacokinetic model.

Previously developed semi-physiological structural pharmacokinetic model (Fig. 1) was adopted to simultaneously describe plasma pharmacokinetics and CNS disposition of ondansetron in WT, KO, and OT groups. In the model, Pgp-mediated transport was only included as an efflux process from the brain and spinal cord compartments to the central distribution compartment. This model structure was supported by the fact that WT and Pgp KO groups did not show any significant difference in ondansetron plasma pharmacokinetics [18]. In this study, concentration of ondansetron was higher, and the half-life was longer after co-administration of ondansetron with tariquidar compared to both WT and KO groups (Figs. 2 and 3). The reason for these changes is not completely understood; however, it can be hypothesized that they were not related to the effect of tariquidar on Pgp, because in animals lacking Pgp (KO groups) no such change was observed. To overcome this, separate terms for central volume of distribution (V_1,TRQ_) and elimination rate constant (k_el,TRQ_) were required to capture the data in OT groups. In the final model a separate term for describing Pgp function in OT groups (k_Pgp,TRQ_) was estimated; and the value was several orders of magnitude lower compared to WT rats, which indicates an almost complete inhibition of Pgp-mediated transport. The rest of the parameters were shared between WT, KO, and OT strains; and parameters were estimated separately for male and female rats. The final model provided good simultaneous description of all tested tissues (plasma, spinal cord, brain, and CSF) in WT, KO, and OT groups for male (Fig. 2) and female (Fig. 3) rats, and all parameters were estimated with sufficient precision (Table III).

**Table III:** Final Model Estimated Parameters for Plasma Pharmacokinetics and CNS Disposition of Ondansetron Male and Female Wild-Type and Pgp Knock-Out Rats Following Administration of Ondansetron Alone and Male and Female Wild-Type Rats Following Co-administration of Ondansetron with Tariquidar

Parameter	Parameter Description	Unit	Male		Female	
			Estimate	CV%	Estimate	CV%
V_1_	Central volume of distribution	mL	17.5	6	13.3	7
V_1,TRQ_	Central volume of distribution	mL	12.7	6	11.3	7
k_el_	Systemic elimination rate constant	h^−1^	3.60	9	5.06	11
k_el,TRQ_	Systemic elimination rate constant for OT animals	h^−1^	3.38	9	3.02	11
k_12_	Distribution rate constants to/from peripheral distribution compartment	h^−1^	2.14	22	4.37	19
k_21_		h^−1^	2.62	9	2.32	10
k_13_	Distribution rate constants to/from brain compartment	h^−1^	17.6	16	13.0	14
k_31_		h^−1^	39.6	16	25.2	18
k_14_	Distribution rate constants to/from spinal cord compartment	h^−1^	9.37	18	3.60	17
k_41_		h^−1^	71.0	18	31.5	22
k_15_	Distribution rate constants to/from CSF compartment	h^−1^	0.081	29	0.518	17
k_51_		h^−1^	6.84	64	47.3	25
k_35=45_	Rate constant for brain/spinal cord and CSF exchange	h^−1^	0.928	21	0.914	18
k_53=54_		h^−1^	66.6	16	79.9	15
k_Pgp_	Rate constant for Pgp-mediated efflux from the CNS	h^−1^	81.9	16	101	19
k_Pgp,TRQ_	Rate constant for Pgp-mediated efflux from the CNS for OT animals	h^−1^	0.002	11	0.0001	59
V_brain_	Brain volume	mL	1.8^*^	-	1.8^*^	-
V_spinal_	Spinal volume	mL	0.6^*^	-	0.6^*^	-
V_CSF_	CSF volume	mL	0.25^*^	-	0.25^*^	-
*var_P*	Variance		0.05	18	0.03	19

^*^: Fixed parameters

**Fig. 6:**
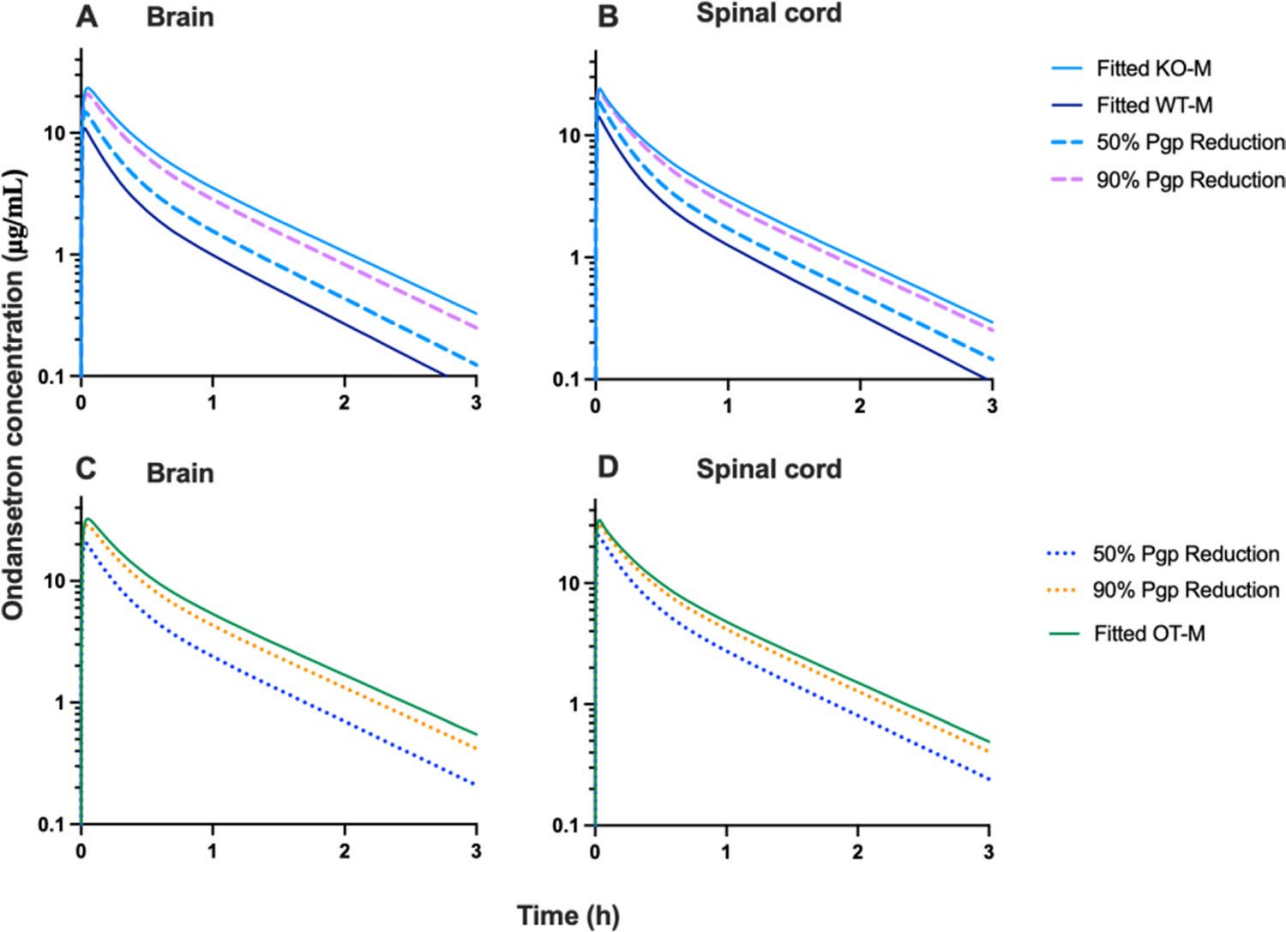
Simulations based on k_Pgp_ reduction in WT-M by 50% and 90% were evaluated and overlaid with the fitted profiles for WT-M and KO-M (**A**, **B**). Simulations based on k_Pgp_ reduction by 50% and 90% (**C**, **D**) were completed in OT-M animals and overlaid with fitted OT-M profile. Panels (**A**) and (**B**) – simulations were performed with systemic disposition parameters for WT; and panels (**C**) and (**D**) simulations were performed with parameters for OT group.

**Fig. 7:**
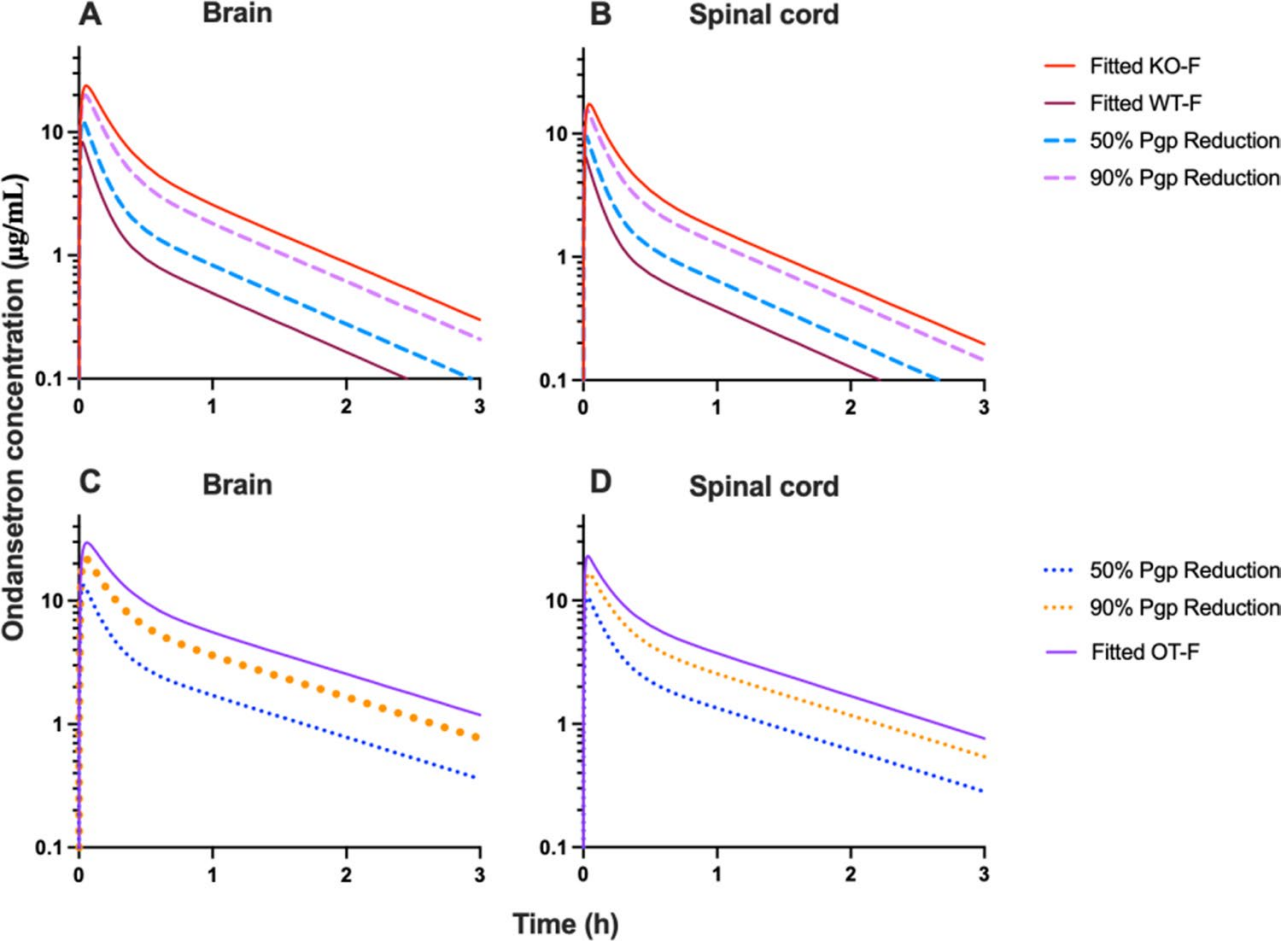
Simulations based on k_Pgp_ reduction in WT-F by 50% and 90% were evaluated and overlaid with the fitted profiles for WT-F and KO-F (**A**, **B**). Simulations based on k_Pgp_ reduction by 50% and 90% (**C**, **D**) were completed in OT-F animals and overlaid with fitted OT-F profile. Panels (**A**) and (**B**) – simulations were performed with systemic disposition parameters for WT; and panels (**C**) and (**D**) simulations were performed with parameters for OT group.

Simulations were conducted to visualize the effect of the extent of Pgp inhibition on ondansetron exposure in the CNS. The effect of tariquidar on plasma pharmacokinetics of ondansetron was considered independent of Pgp function. Two sets of simulations were performed using parameters for either WT or OT groups. The value of k_Pgp_ estimated for WT group was decreased by 50% or 90%. Figures 6 and 7 show simulated profiles for ondansetron exposure in brain and spinal cord in male and female rats: panels A and B – simulations with systemic disposition parameters for WT; and panels C and D simulations with parameters for OT group. Model fits for WT, KO, and OT are presented for comparison. For example, 1 h after ondansetron dose, 50% and 90% of Pgp inhibition would result in 1.5- and 3-fold higher concentration in the brain on male rats; and 1.2- and 2-fold in the spinal cord, respectively.

## Discussion

In a previous work, we have shown that ondansetron distribution to various parts of the CNS is affected by a genetic knockout of Pgp efflux transporter [18]. In the current study, we evaluated the utility of co-administering ondansetron with tariquidar (a third generation Pgp inhibitor) in wild type animals to enhance exposure of the CNS to ondansetron. In both male and female rats, tariquidar demonstrated effective inhibition of Pgp efflux; and ondansetron disposition to the CNS tissues was significantly increased compared to wild-type animals receiving ondansetron alone (Figs. 2 and 3). The extent of tariquidar effect at the level of CNS was comparable to the effect of genetic knock out of the Pgp transporter. Interestingly, the plasma disposition was also altered when ondansetron was co-administered with tariquidar (as discussed below). The model (Fig. 1) successfully captured plasma and all CNS tissue profiles simultaneously in OT groups (from this study) and previous data from WT and KO groups [18], and all parameters were estimated with sufficient precision. Observed data and modeling suggest that complete inhibition of Pgp and the CNS was obtained with tariquidar (Figs. 2 and 3 and Table III). We also showed that tariquidar concentrations in plasma, brain, and spinal cord did not significantly change during the course of the experiment (Fig. 5); this trend was also observed in our previous work [20]. Due to this prolonged half-life of tariquidar, a sigmoid inhibitory function that was originally proposed for describing Pgp inhibition in OT groups or the use of brain/spinal cord tariquidar concentration could not be included in the model; and the final model incorporated tariquidar administration as a categorical covariate. Simulations were performed to assess potential effect of a partial Pgp inhibition on ondansetron partition to the brain and spinal cord (Figs. 6 and 7), and this can be further evaluated in future studies.

In this proof of concept study, a relatively high dose of tariquidar was used with a purpose to demonstrate the maximum potential enhancement in CNS distribution of ondansetron in comparison to ondansetron injected alone. Previously, a complete inhibition of Pgp was reported 30 min after intravenous injection of tariquidar doses above 6 mg/kg [19]. A dose of 15 mg/kg was used in several preclinical reports evaluating Pgp inhibition by tariquidar; therefore, we initially tried a dose of 15 mg/kg of tariquidar administered 30 min before ondansetron (10 mg/kg) in our study. However, this dose level of tariquidar led to toxicity in rats immediately after ondansetron injection. The toxicity was not observed before administration of ondansetron. Decreasing tariquidar dose to 7.5 mg/kg allowed for eliminating this toxicity, and this dose level was selected for future experiments.

Co-administration of ondansetron (10 mg/kg) with tariquidar to wild type rats resulted in almost doubled plasma ondansetron concentration in comparison to ondansetron administered alone to wild-type (and Pgp KO) rats. Previously, we observed a similar phenomenon when administering ondansetron intravenously (5 mg/kg) with tariquidar (15 mg/kg) in which case the plasma concentration of ondansetron was very similar to ondansetron 10 mg/kg injected alone [18, 27]. Due to the fact that concentrations of ondansetron in OT groups were higher than in KO groups (lacking Pgp), it could be hypothesized that this finding was not directly related to inhibition of Pgp by tariquidar and that some other tariquidar-induced changes occurred. The effect on plasma disposition of drugs due to co-administration of tariquidar has been reported before [27–29]. For example, significantly higher plasma AUC_0-∞_ and lower CL was observed for intravenous ciprofloxacin (7 mg/kg) in male Wistar rats co-administered with 15 mg/kg of tariquidar [27]. On the other hand, the no effect of tariquidar on plasma pharmacokinetics was observed for loperamide or in another study with ciprofloxacin [30, 31]. Another relatively selective third generation Pgp inhibitor, elacridar, was shown to affect cytochrome P450 enzyme function [32]. Separate parameters for ondansetron elimination (k_el,TRQ_) and central volume of distribution (V_1,TRQ_) were required to capture plasma profiles in OT groups. Overall, experimental results of this study (also supported by modeling) suggest that a higher CNS exposure to ondansetron in OT groups relative to KO groups is potentially related to a higher ondansetron exposure in the plasma that drives partitioning to the CNS.

 Previous works reported sex-dependent differences in plasma disposition of ondansetron between males and females, both preclinically and clinically [33, 34]. In our previous work, we found some minor sex-dependent differences in ondansetron pharmacokinetics and CNS disposition in both WT and Pgp KO rats [18]. In this study, a more pronounced difference in plasma and CNS profiles of ondansetron was observed between OT and KO groups in female rats in comparison to males (Figs. 2 and 3). It is currently unclear whether these differences are due to the Pgp-unrelated effects of tariquidar on ondansetron pharmacokinetics.

## Conclusion

In conclusion, the study provided important quantitative information on the role of Pgp in limiting ondansetron exposure in various regions of the CNS using tariquidar (a Pgp inhibitor) in wild type rats. Noncompartmental analysis of the data and modeling results demonstrate that tariquidar at 7.5 mg/kg resulted in an almost complete inhibition of Pgp efflux of ondansetron in the brain and spinal cord. Our results also highlighted the effect of tariquidar on plasma disposition of ondansetron, which may not be dependent on Pgp inhibition, and should be evaluated in future studies. The model successfully captured pharmacokinetics of ondansetron in wild type and Pgp KO animals receiving the drug alone or in wild type animals receiving the ondansetron and tariquidar combination. Our work provided important evidence for utilizing tariquidar to evaluate enhanced CNS disposition of ondansetron (and potentially other Pgp substrates) in the clinic to achieve therapeutic drug concentrations of drugs that may provide successful treatment for neuropathic pain. Clinical study is currently ongoing, and results will become available in the future.

## Abbreviations

5HT: Serotonin
BBB: Blood brain barrier
CNS: Central nervous system
CSF: Cerebrospinal fluid
KO-F: Pgp knockout female rats
KO-M: Pgp knockout male rats
OT-F: Wild type female rats that received a combination of ondansetron and tariquidar
OT-M: Wild type male rats that received a combination of ondansetron and tariquidar
Pgp: P-glycoprotein
WT-F: Wild type female rats
WT-M: Wild type male rats
